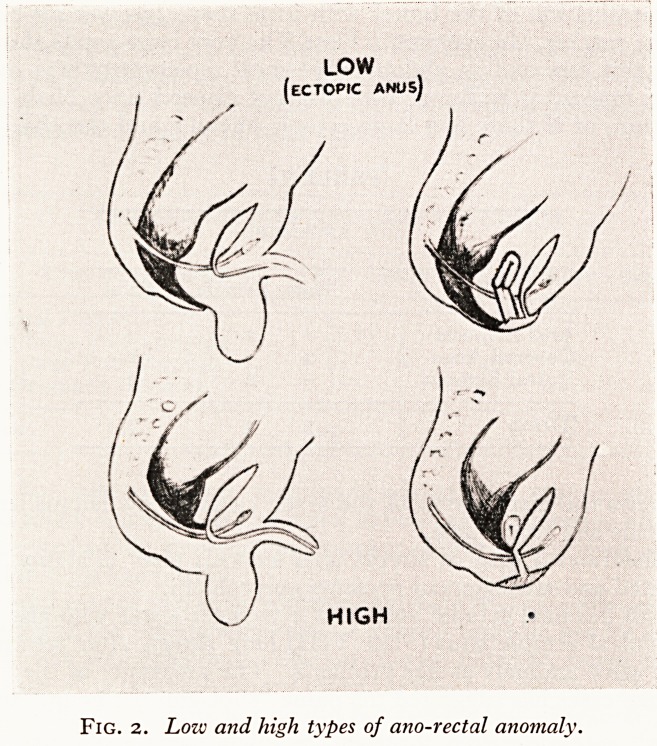# Congenital Ano-Rectal Anomalies
*A paper read to the South-West Paediatric Club.


**Published:** 1963-04

**Authors:** A. G. McPherson

**Affiliations:** Consultant Surgeon, Southmead Hospital, Bristol


### CONGENITAL ANO-RECTAL ANOMALIES*

BY

A. G. MCPHERSON, M.CHIR., F.R.C.S.

*Consultant Surgeon, Southmead Hospital, Bristol*

My subject is congenital ano-rectal anomalies, commonly but inaccurately called imperforate anus, but in a short paper it would be impossible to deal comprehensively with the subject so I propose therefore to concentrate on its clinical aspects.

The material is provided by a personal series of 18 cases referred to me since 1957 at Southmead Hospital, by my Paediatric colleagues Professor A. V. Neale and Dr. Beryl Corner.

My first point of emphasis is that a classification to be of practical value must separate clearly the two distinct groups of anomaly, the low anomalies and the high anomalies. Low anomalies are correctable by a very simple procedure and will be followed by anal continence approaching the normal. High anomalies, on the other hand, require a major operative procedure and will have anal function which is grossly imperfect. In this series there are 7 low and 11 high anomalies, only 3 of the latter being in females.

The classification of Ladd and Gross is widely recognised but does not underline this important division of cases. Ladd's Type I, anal stenosis, is relatively uncommon; I have 3 cases. Ladd's Type II, anal membrane, and Ladd's Type IV, are very rare; I have no such cases. Ladd's Type III (Fig. 1) provides the bulk of the cases and Figs. 1 and 2 indicate my second point of emphasis that the majority of these cases have a fistula. In fact, when no anal opening is present in the normal position one must expect an ectopic opening. In the male this is likely to be into the urinary tract (the upper two arrows), in the female into the vagina or vulva or anterior to the normal site in the perineum in either sex.

FIG. 1. *Ladd's type III anomaly showing various possible sites of fistula.*

*A paper read to the South-West Paediatric Club.

PLATE V.

*X-ray in inverted position: low anomaly.*

PLATE VI.

*X-ray in inverted position: high anomaly.*

PLATE VII. *Anal stenosis.*

PLATE VIII. *Vulval ectopic anus.*

PLATE IX.
*High anomaly in the male (post-mortem specimen).*

PLATE X.
*High anomaly in the female with cloaca (recto-vaginal fistula), (post-mortem specimen).*

PLATE XI. *High anomaly, before treatment.*

PLATE XII. *High anomaly, after treatment.*

PLATE XIII. *High anomaly with cloaca, before treatment.*

PLATE XIV. *High anomaly with cloaca, early post-operative result.*

FIG. 2. *Low and high types of ano-rectal anomaly.*

Figure 2 demonstrates the next important point of emphasis, namely that when the ectopic opening is low, as in the upper diagrams, one expects to find the rectum low, whereas with high openings there is an associated agenesis of the rectum and the bowel terminates above the levator ani sling.

Associated anomalies are listed in Table I from which it will be seen that a fistula into the lower urinary tract is very common in these cases. All the fistulae occurred in the high group. Other anomalies were uncommon in this particular series, but were the direct or contributory cause of death in some of the fatal cases.

TABLE I

ASSOCIATED ANOMALIES

| | |
|---|---|
| Fistula into Urinary Tract .. | 9 (including 2 cloaca) |
| Colon Agenesis, Malrotation .. | 1 ,, |
| Sacral Agenesis .. .. .. | 1 ,, |
| Hypospadias and Bifid Scrotum | 1 ,, |
| Meningomyelocoele .. .. | 1 ,, |

*Diagnosis:*

Early diagnosis of an ano-rectal anomaly follows adequate routine examination of the newborn. The recognition of the level is not always so simple. A bulging perineum suggests a low anomaly. Careful examination in a good light may disclose a minute opening not at first obvious, which may be at the anal site or, as mentioned above, anteriorly placed.

A plain lateral X-ray in the inverted position is often helpful. Plate V shows a very obvious low termination of the bowel with little tissue intervening between the gas shadow and the marker. In contrast in Plate VI a very large gap is shown. The anus was absent in this case and an abdomino-perineal replacement was required. X-ray may, however, mislead if sufficient time has not elapsed since birth to allow gas to reach the rectum, or if there is a large ectopic opening and the child is not acutely obstructed.

TABLE II

LOW ANOMALIES

| Type | Male | Female | Total |
|---|---|---|---|
| Anal Stenosis .. | 1 | 2 | 3 |
| Covered Anus .. | 2 | – | 2 |
| Vulval Ectopic .. | – | 2 | 2 |
| TOTAL .. .. | 3 | 4 | 7 |

*Low anomalies:*

Table II shows the distribution of the seven cases of low anomalies in the series, four being female and three male.

Plate VII illustrates a case of simple anal stenosis after dilatation. The anus is normally situated and management presents no problem.

One boy with perineal ectopic anus had a younger sister who also figures in the series with a vulval ectopic anus (Plate VIII) here shown after treatment. This is essentially the same anomaly as her brother's. The proximity of the vulval and anal openings in such cases has suggested the name "shot-gun perineum" used by Dennis Browne.

All the cases of low anomaly, with the exception of one case of anal stenosis in a baby with meningo-myelocoele who died, have done very well following very simple procedures. Continence appears excellent.

*High anomalies:*

High anomalies present a much more serious problem. Plate IX (from a specimen in Southmead Hospital museum—not one of my series) shows the common type of high anomaly in the male. The bowel stops above the pelvic diaphragm, a fistula is usually present in the region of the posterior urethra. Plate X shows the state of affairs in the female in one of the two cases of cloaca in this series. Again, the bowel stops high and a fistula is clearly shown entering the cloaca below the bladder neck. The uterus which is bicornuate does not intervene between bowel and bladder. Plate XI shows the external appearance.

Table III shows the distribution of the 11 cases of high anomaly. 8 cases were in males; only 3 cases including the two of cloaca were female.

TABLE III

HIGH ANOMALIES

| Type | Male | Female | Total |
|---|---|---|---|
| Ano-rectal Agenesis .. | 7 | 1 | 8 |
| Ano-rectal Stenosis .. | 1 | – | 1 |
| Cloaca .. .. | – | 2 | 2 |
| TOTAL .. .. | 8 | 3 | 11 |

*Results in cases of high anomaly:*

Table IV shows the treatment and results in this much more serious group. One case had a perineal dissection, two cases had a two-stage abdomino-perineal replacement and seven cases had a one-stage abdomino-perineal replacement, which I regard as the operation of choice in most cases.

TABLE IV

TREATMENT OF HIGH ANOMALIES AND RESULTS

| Type of Operation | No. | Male | Female | Alive | Dead |
|---|---|---|---|---|---|
| Perineal Dissection .. .. | 1 | – | 1 | – | 1 |
| Two Stage Pull Through .. | 2 | 2 | – | 1 | 1 |
| One Stage Pull Through .. | 7 | 6 | 1 | 5 | 2 |
| No Operation (Cloaca) .. | 1 | – | 1 | – | 1 |
| TOTAL .. .. .. | 11 | 8 | 3 | 6 | 5 |

In addition, the case of cloaca mentioned above who was premature and had flaccid legs and sacral agenesis, also, was not submitted to operation and died.

A single case treated by perineal replacement unfortunately died at three weeks from an intractable pyelonephritis.

In the successful case of two stage replacement there was no evidence of a fistula to the urinary tract and the second stage was postponed until he was 18 months of age, when operation was well tolerated. The other case failed to thrive after an initial colostomy because of persistent urinary infection due to a fistula. The second stage was pressed on with at 3 months but he did not survive this operation.

Seven cases had a one stage abdomino-perineal replacement or "pull-through." There were two deaths. The first case in the series died shortly after operation, the second death was a case with total colon agenesis, the fistula in this case being into the bladder. This case who had an ileal "pull-through" proved difficult to maintain in fluid and electrolytic balance. His buttocks became excoriated from the profuse ileal discharge and he went down hill and died at 3 weeks.

The other 5 of the 7 cases of one stage "pull-through" have survived; this included the last four consecutive cases. Plates XI and XII show one such case. A limited perineal exploration excluded a low level rectum, the usual fistula was found at abdominal exploration and disconnected. It is an essential part of the after care of these cases to dilate the anus regularly until all tendency to stenosis has ceased.

The latest case to be submitted to one stage "pull-through" (Plates XIII and XIV) was the second cloaca in the series. The operative findings were similar to the post-mortem findings of the earlier case described above. This baby was not obstructed and passed both urine and faeces through the single channel which served as urethra, vagina and anal canal. This child has some form of bladder neck and clinical observation, cystoscopy, and pyelography all suggest that she will have continence of urine.

TABLE V

TREATMENT OF HIGH ANOMALIES AND OPERATIVE RESULTS

| Type of Operation | No. | Alive | Dead | Cause |
|---|---|---|---|---|
| Perineal Dissection .. | 1 | – | 1 | Pyelonephritis |
| Two Stage Pull Through | 2 | 1 | 1 | Operation |
| One Stage Pull Through .. | 7 | 5 | 2 | Operation<br>Bowel insufficiency |

Table V summarises the results in the operated cases of high anomalies. Most cases have been submitted to the one stage operation which I favour for the following reasons. The fistula into the urinary tract is at once disconnected, the operation is unhindered by a previous colostomy, and the difficulties of colostomy management in infancy, which I find considerable, are avoided. These infants tolerate the procedure well, provided one has expert anaesthetic and paediatric assistance. The importance of the anaesthetist's contribution cannot be over-estimated. With modern anaesthetic technique these babies now leave the operating table in excellent condition. Briefly, the anaesthetic requirements are good relaxation with minimal narcosis and adequate ventilation. This demands intratracheal intubation. A stomach tube is passed routinely before operation and a reliable cut-down intravenous drip should be in position before operation. Blood is replaced as lost and the baby should not become chilled during the operation.

Operative detail will not be gone into here. Post-operatively intravenous fluids and gastric aspiration are continued until feeds, which are started at 24 hours and progress in "ladder" fashion, are being assimilated.

*Summary;*

My conclusions from this small personal series are as follows:

1. Accurate assessment of the type of anomaly is the key to treatment.
2. An ectopic opening is to be expected in the majority.
3. Meticulous examination of the perineum and genitalia may disclose a tiny low opening which may be exploited.
4. Low anomalies are easy to treat and the results verge on the normal.
5. High anomalies usually require abdomino-perineal replacement and a one stage procedure is to be favoured.
6. Normal anal continence is not to be expected in case of high anomaly.

I think these babies are well worth salvage and that major surgical intervention is fully justified.

Finally I should like to correct any false impression that I may have given that I am only interested in the infant as an anus. On the contrary, I am interested in him as a whole!

In conclusion, may I thank my paediatric colleagues for their help and interest in this series, also Dr. G. R. Airth who was responsible for the radiographs, Mr. W. Sweet who took the photographs and Dr. N. J. Brown who provided the post-mortem specimens.

## Figures and Tables

**Fig. 1. f1:**
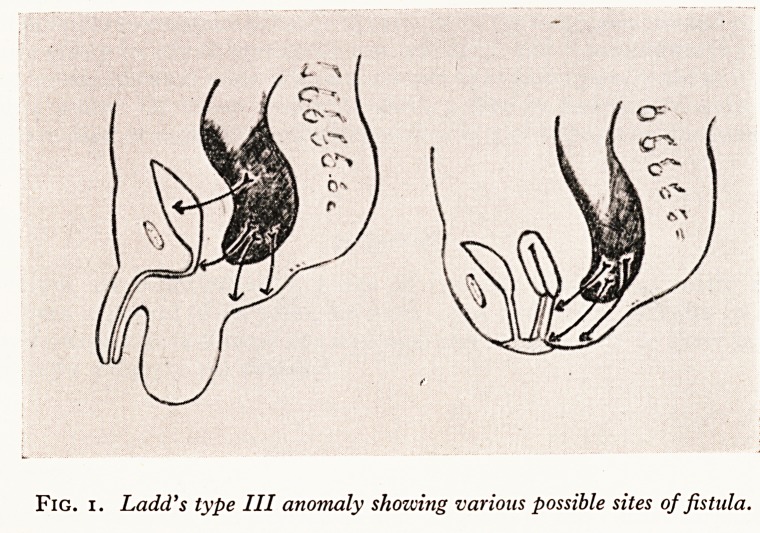


**PLATE V. f2:**
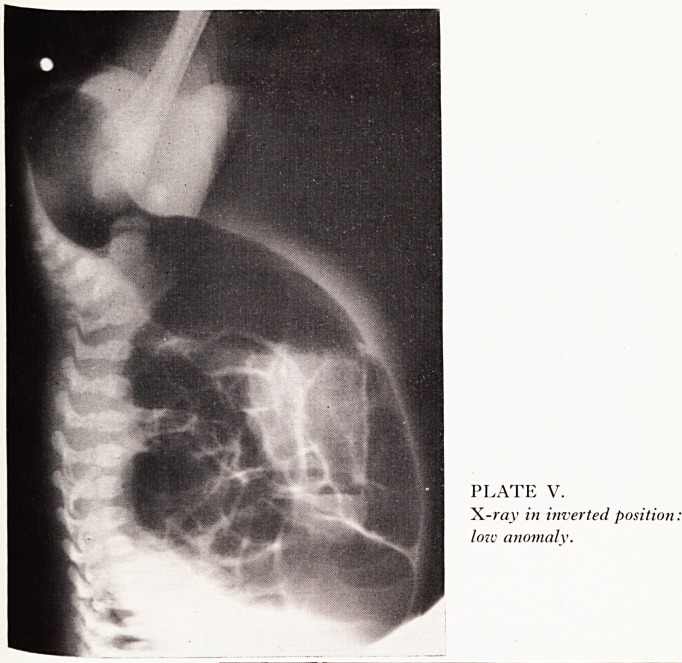


**PLATE VI. f3:**
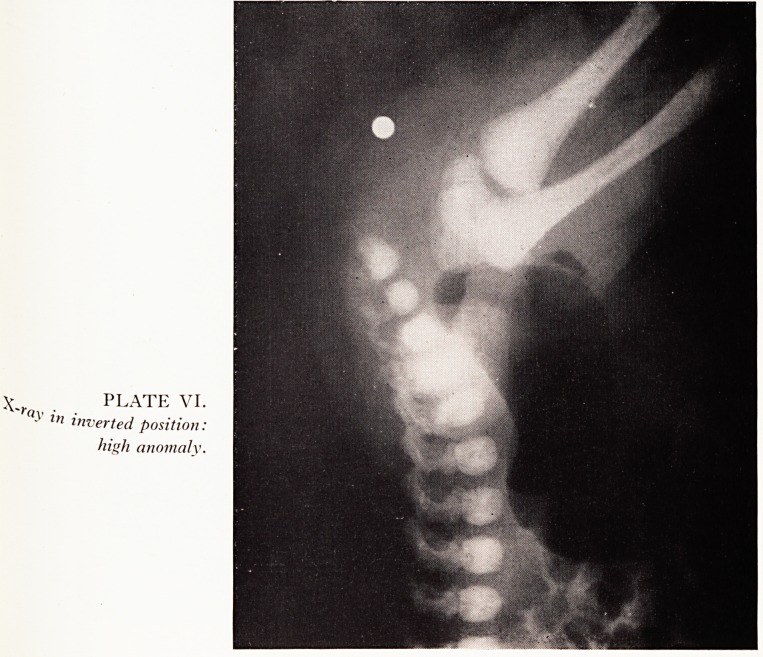


**PLATE VII. f4:**
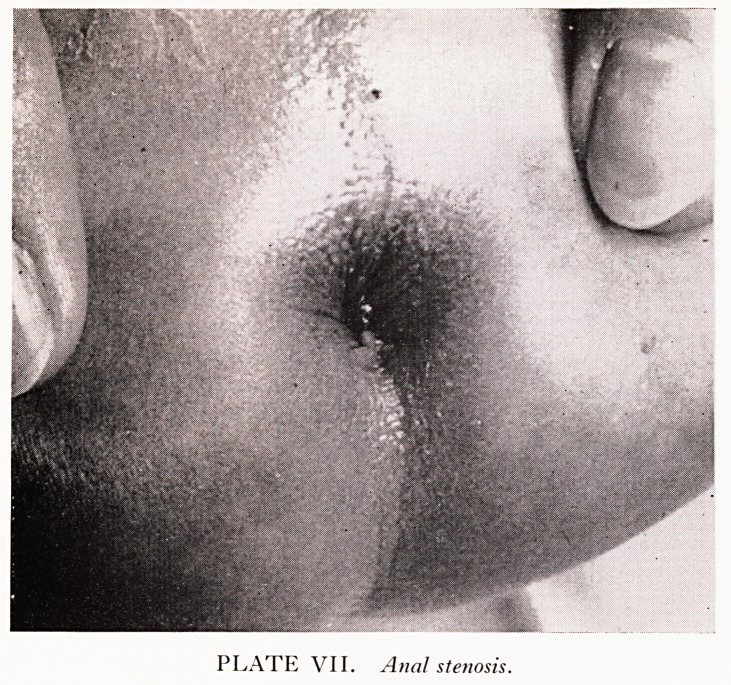


**PLATE VIII. f5:**
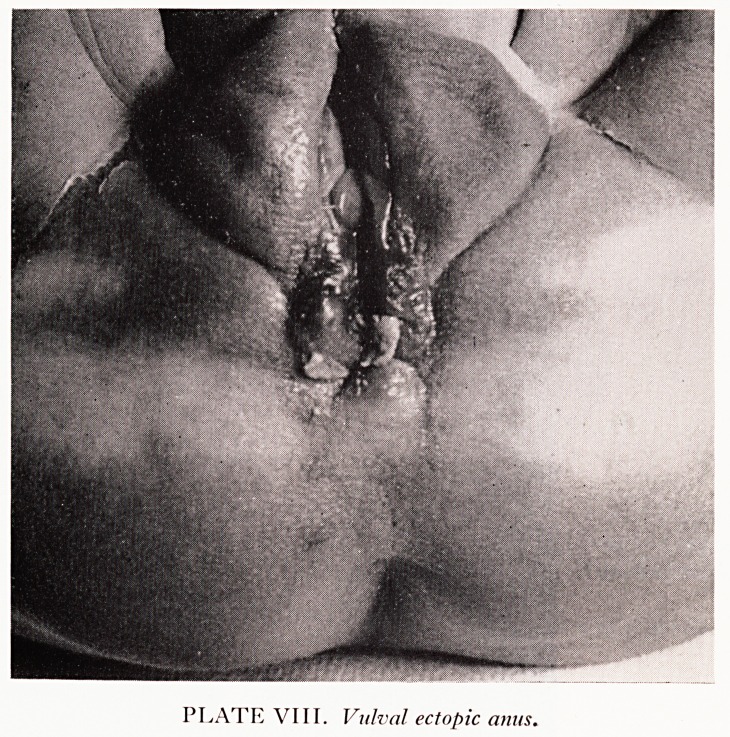


**PLATE IX. f6:**
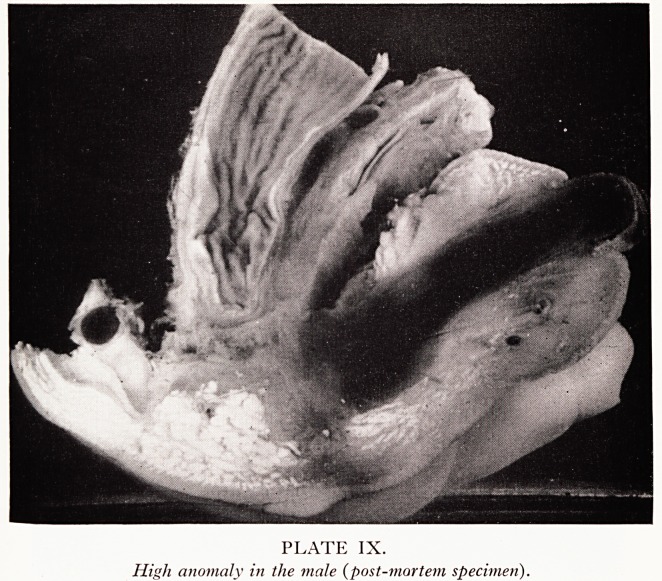


**PLATE X. f7:**
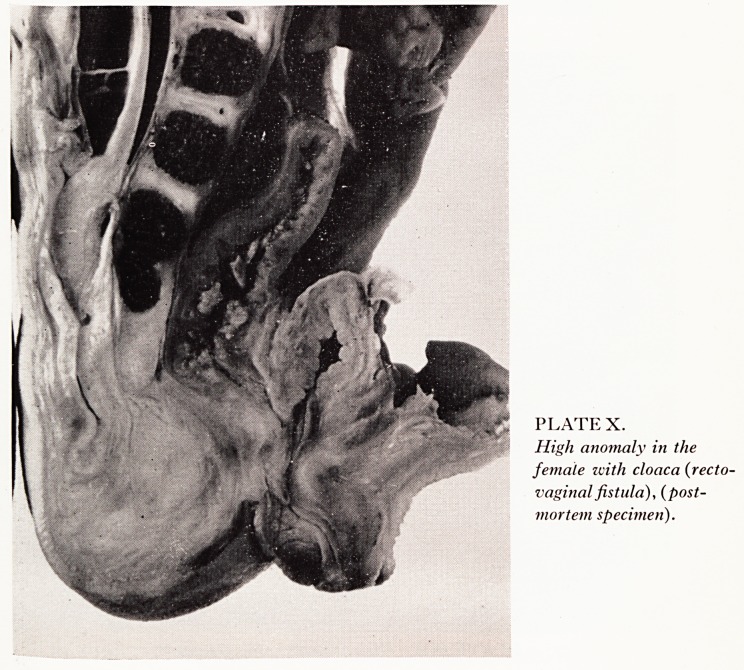


**PLATE XI. f8:**
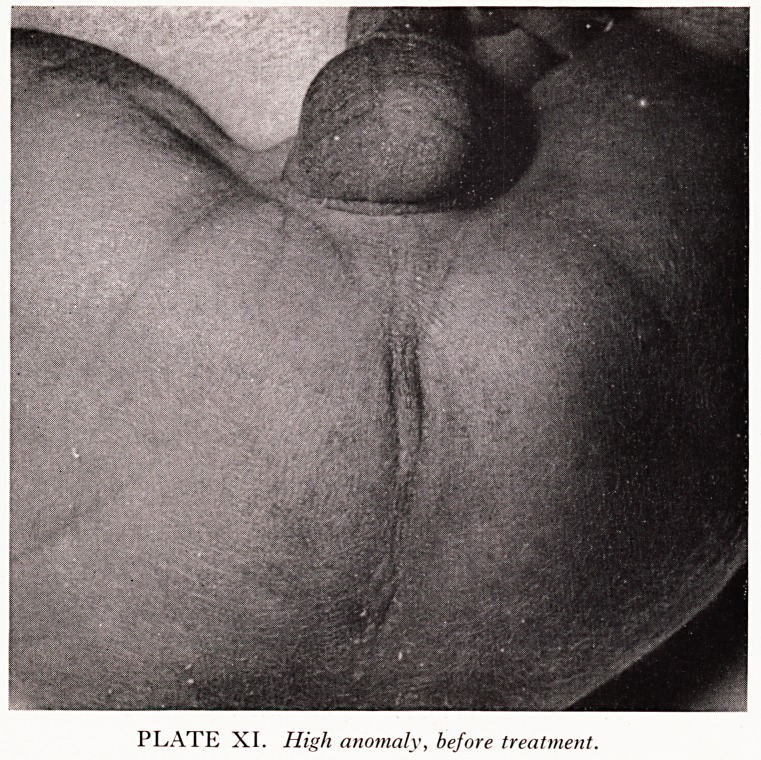


**PLATE XII. f9:**
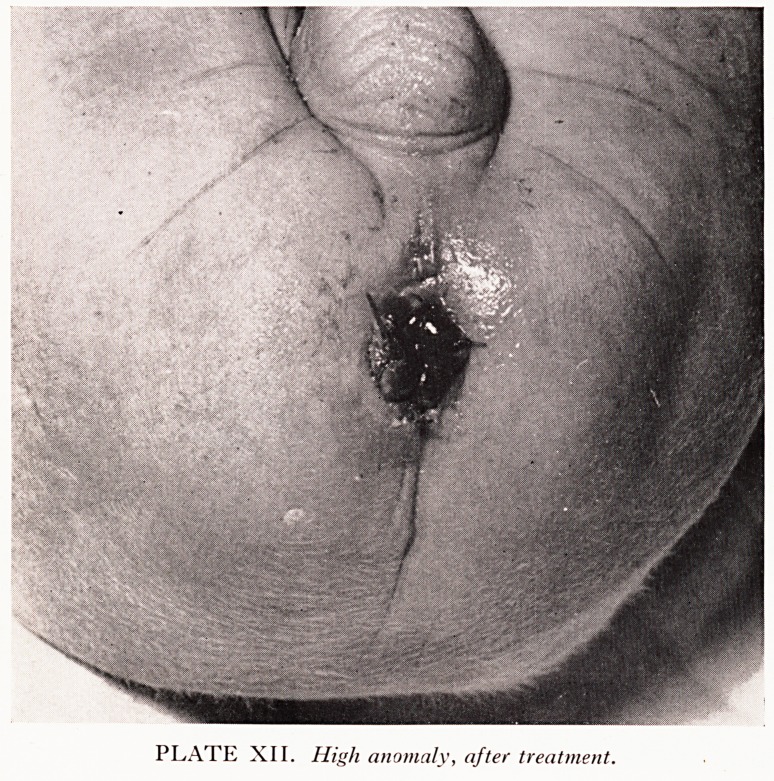


**PLATE XIII. f10:**
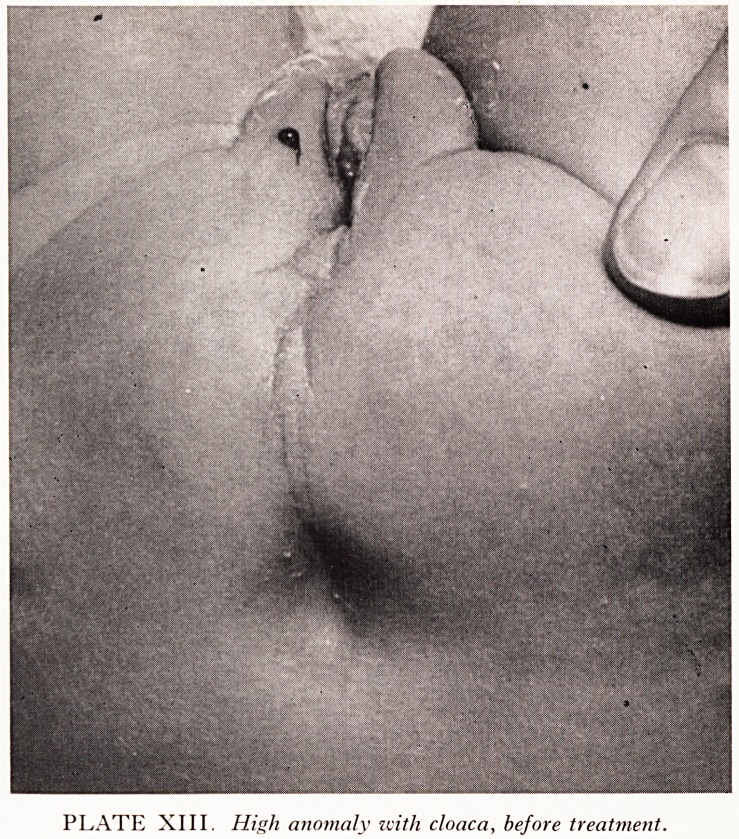


**PLATE XIV. f11:**
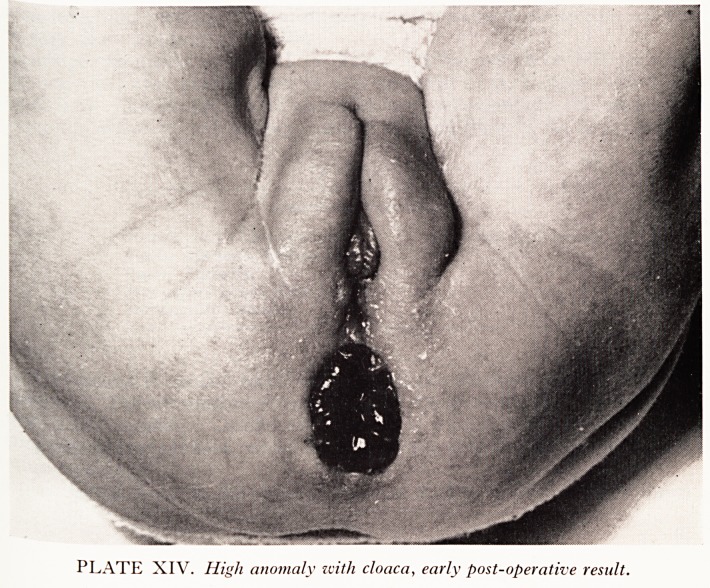


**Fig. 2. f12:**